# Editorial: Use of artificial intelligence to improve maternal and neonatal health in low-resource settings

**DOI:** 10.3389/fgwh.2025.1675578

**Published:** 2025-09-02

**Authors:** Z. Hoodbhoy, S. Martinez-Sanchez, M. I. Nisar, E. Smith

**Affiliations:** 1 Department of Paediatrics and Child Health, The Aga Khan University, Karachi, Pakistan; 2 PredictBy Research and Consulting, Barcelona, Spain; 3 Department of Engineering, Universitat Pompeu Fabra, Barcelona, Spain; 4 Milken School of Public Health, George Washington University, Washington, DC, United States

**Keywords:** artificial intelligence, maternal health, neonatal health, risk stratification, low middle income countries

The wide disparities in maternal and neonatal morbidity and mortality, both within and between countries, reflect inequities in access to quality care as well as the unequal distribution of risk factors for adverse pregnancy outcomes (1). Use of technology such as artificial intelligence (AI) can be leveraged for risk stratification and improved clinical decision support especially in Low-middle income countries (LMICs) (2). This editorial highlights the role of AI for prevention, prediction, diagnosis, or management of maternal and neonatal health conditions, with a particular focus on low resource settings. There are four studies that are published in this issue. These include the application of AI for automated segmentation of ultrasound images, development of algorithms using labor and delivery data from Africa, prediction models for postpartum hemorrhage (PPH), and a meta-analysis on gestational age (GA) assessment.

Assessment of placental and fetal health using Doppler imaging is a routine practice during obstetric care. Aguado et al. proposed a deep learning approach to image analysis for the umbilical artery (UA), middle cerebral artery (MCA) and left ventricular inflow and outflow (LVIO) Dopplers to ensure standardization across users. Using an imaging dataset from Pakistan and Spain, the authors reported >85% accuracy on the UA and MCA Dopplers but considerably lower on the LVIO images. Beyond image classification, their AI models were also able to extract essential clinical measurements, offering a streamlined, automated workflow. This AI enabled workflow offers a potential solution for reducing operator dependence and ensuring consistent interpretation of images during routine care.

Nogueira et al. utilized an unsupervised machine learning approach on labor and delivery data acquired from the BOLD study, which was conducted in Nigeria and Uganda (3). The authors utilized labor and delivery data from approximately 10,000 women to develop a prototype which serves as a real-time decision-support tool for assisting care providers in risk assessment during labor and appropriate intervention. Unlike the traditional WHO partograph, which offers limited adaptability and has shown limited evidence of impact, the proposed method leverages machine learning to enable more accurate, context-specific clinical decisions. These personalized insights are especially valuable in LMICs where shortages of skilled healthcare providers may result in lack of identification of danger signs during labor and lead to avoidable interventions such as caesarean sections.

The study by Shah et al. utilized antenatal and labor and delivery data collected from a prospective pregnancy cohort in Kenya to predict PPH. Among the four AI models tested, the naïve Bayes model performed best on the test set with an area under the curve of 0.76 to predict PPH. This model identified seven key predictors for PPH including anemia during pregnancy, signs of pallor during antenatal care (ANC), limited number of ANC visits along with abnormal blood pressure during the intrapartum phase (Shah et al.). Models such as the one proposed could support early risk stratification for timely management, potentially reducing the incidence of life-threatening outcomes like PPH.

The diagnostic accuracy meta-analysis by Naz et al. assessed the reliability of AI models on assessing GA on 2 dimensional as well as blind sweep images as compared to conventional ultrasound. The algorithms demonstrated high accuracy, with mean errors of 4.3 days for 2D ultrasound images and 2.5 days for blind sweep videos, highlighting their potential for reliable gestational age estimation. These findings highlight the potential of AI in pregnancy dating, particularly in resource-limited settings where access to trained sonographers is scarce.

Collectively, these studies underscore the potential of AI in maternal and neonatal health, from automating GA assessment to enabling personalized risk prediction for cesarean section and PPH. In LMICs, where provider-to-population ratios are low, ensuring access to quality care remains a pressing challenge. Evidence shows that AI algorithms can accurately estimate GA from ultrasound images and extract critical information from fetal Dopplers. As the World Health Organization recommends that all women receive at least one scan before 24 weeks of GA (4), leveraging technology and AI could help bridge this gap and improve timely access to essential obstetric care. Similarly, conditions like hemorrhage continue to be a leading cause of maternal morbidity and mortality (5). Identifying easily obtainable predictors through AI could enable healthcare providers to predict PPH and adopt timely preventive strategies. In summary, the data presented in this Research Topic adds to the evidence that AI-driven risk stratification offers a promising pathway toward more proactive rather than reactive maternal care.

As AI algorithms continue to evolve, ensuring transparency and regulatory compliance will be essential for large scale deployment. Using the Paul Farmer's framework, McCoy et al. have proposed using the 5S framework (*staff, stuff, space, systems and support*) for AI deployment in LMICs (6). This framework emphasizes the need for adequately trained personnel (staff), resources including tools and technologies (stuff), enabling physical and digital infrastructure (space), efficient processes and governance structures (systems), and ongoing technical, financial, and policy backing (support). Applying such a structured approach could help ensure that AI technologies are not only technically sound but also operationally feasible, thus ensuring integration into healthcare delivery in resource-limited settings.

The manuscripts in this section strengthen the evidence that AI can both optimize care and enable timely risk stratification both of which can be potential game changers in low-resource settings. The challenge now lies in deploying these algorithms equitably to bring us closer to the elimination of preventable maternal and newborn deaths.
